# Principles of Forensic Medicine

**Published:** 1845-06

**Authors:** 


Art. VII.—*Principles of Forensic Medicine.* By William A. Guy, M.B. Cantab., Fellow of the Royal College of Physicians; Professor of Forensic Medicine, King's College, London; Physician to the King's College Hospital, &c., &c., &c. First American Edition. With Notes and Additions, by Charles A. Lee, M.D., Professor of Materia Medica and General Pathology in Geneva College, &c., &c. N. York: Harper & Brothers. 1845. 8vo. pp. 711.

This volume is a rich repository of facts, to which the student of medical jurisprudence, and the practitioners of medicine and law may have recourse with equal advantage. Its value to the profession in this country has been very materially enhanced by the additions of Professor Lee. As a work of labor and research it cannot pretend to compete with the "Elements" of our countrymen, the Drs. Beck, but its merits as a manual are not the less on that account. It is concise, lucid, and practical, with sufficient details to make the principles clear, without burdening the memory of the reader. The plan of the author in treating the various topics, is to begin with a short statement of the existing provisions and requirements of the law in England, to which Dr. Lee has added whatever is peculiar in the jurisprudence of this country relative to the subjects discussed. Having stated what the law is, he next takes up and investigates the chief medical questions likely to arise out of it, and under each head gives the necessary practical rules for medico-legal examination. Altogether, the work is an excellent epitome of all that has been established in medical jurisprudence, and will be found an instructive and entertaining volume for any gentleman of taste, whether engaged or not in either of the professions for which it was written.

This is the second republication in this country on the subject of Medical Jurisprudence within the present year, the treatise of Mr. Alfred Taylor having appeared in Philadelphia simultaneously with the work of Dr. Guy in New York. We commended the former of these publications to the favorable regard of our readers sometime ago. Since we wrote that notice, we have seen that Mr. Taylor has charged Dr. Guy with having borrowed from his work without suitable acknowledgments, which has given rise to some bitter sparring between these authors. This is to be regretted. Guy, it seems, was the pupil of Taylor, and if he brought away from the lectures the thoughts, and often the words, of his instructor, it is no more than was to have been expected. But he has acknowledged in general terms his obligations to Mr. Taylor, and that, it occurs to us, ought to have satisfied him. Both works are largely indebted to previous publications, among the rest to the American volumes to which we have already referred. Our countrymen have quite as much cause to quarrel with Mr. Taylor, as Mr. Taylor has to quarrel with Dr. Guy.

